# Uncovering NK cell sabotage in gut diseases via single cell transcriptomics

**DOI:** 10.1371/journal.pone.0315981

**Published:** 2025-01-03

**Authors:** Hansong Lee, Dai Sik Ko, Hye Jin Heo, Seung Eun Baek, Eun Kyoung Kim, Eun Jung Kwon, Junho Kang, Yeuni Yu, Ninib Baryawno, Kihun Kim, Dongjun Lee, Yun Hak Kim

**Affiliations:** 1 Medical Research Institute, Pusan National University, Yangsan, Republic of Korea; 2 Division of Vascular Surgery, Department of General Surgery, Gachon University College of Medicine, Gil Medical Center, Incheon, Republic of Korea; 3 Department of Anatomy, School of Medicine, Pusan National University, Yangsan, Republic of Korea; 4 Department of Research, Keimyung University Dongsan Medical Center, Daegu, Republic of Korea; 5 Childhood Cancer Research Unit, Department of Women’s and Children’s Health, Karolinska Institutet, Stockholm, Sweden; 6 Department of Biomedical Informatics, School of Medicine, Pusan National University, Yangsan, Republic of Korea; 7 Department of Convergence Medicine, School of Medicine, Pusan National University, Yangsan, Republic of Korea; 8 Transplantation Research Center, Research Institute for Convergence of Biomedical Science and Technology, Pusan National University Yangsan Hospital, Yangsan, Republic of Korea; Rutgers: Rutgers The State University of New Jersey, UNITED STATES OF AMERICA

## Abstract

The identification of immune environments and cellular interactions in the colon microenvironment is essential for understanding the mechanisms of chronic inflammatory disease. Despite occurring in the same organ, there is a significant gap in understanding the pathophysiology of ulcerative colitis (UC) and colorectal cancer (CRC). Our study aims to address the distinct immunopathological response of UC and CRC. Using single-cell RNA sequencing datasets, we analyzed the profiles of immune cells in colorectal tissues obtained from healthy donors, UC patients, and CRC patients. The colon tissues from patients and healthy participants were visualized by immunostaining followed by laser confocal microscopy for select targets. Natural killer (NK) cells from UC patients on medication showed reduced cytotoxicity compared to those from healthy individuals. Nonetheless, a UC-specific pathway called the BAG6-NCR3 axis led to higher levels of inflammatory cytokines and increased the cytotoxicity of NCR3+ NK cells, thereby contributing to the persistence of colitis. In the context of colorectal cancer (CRC), both NK cells and CD8+ T cells exhibited significant changes in cytotoxicity and exhaustion. The GALECTIN-9 (LGALS9)-HAVCR2 axis was identified as one of the CRC-specific pathways. Within this pathway, NK cells solely communicated with myeloid cells under CRC conditions. HAVCR2+ NK cells from CRC patients suppressed NK cell-mediated cytotoxicity, indicating a reduction in immune surveillance. Overall, we elucidated the comprehensive UC and CRC immune microenvironments and NK cell-mediated immune responses. Our findings can aid in selecting therapeutic targets that increase the efficacy of immunotherapy.

## Introduction

The colon, a vital part of the digestive system, plays a crucial role in absorbing water and nutrients. However, in recent years, the occurrence of colon diseases, including irritable bowel syndrome, inflammatory bowel disease (IBD) and colorectal cancer (CRC), has increased [1–3]. Among the diseases, CRC stands out as the second-most and third-most common cancer among women and men worldwide, respectively [4], and accounts for approximately 10% of cancer-related deaths [5]. It can develop in any part of the large intestine, encompassing the cecum, colon, and rectum [6]. Similarly, the incidence of IBD is continuously increasing worldwide. Approximately 1 million individuals in the United States and approximately 2.5 million in Europe are estimated to be affected by IBD [7]. Among the various types of IBD, ulcerative colitis (UC) is particularly common and widely recognized. UC is characterized by continuous inflammation without skip lesions and is confined to the colon and rectum, overlapping with the regions affected by CRC [8].

The causes of UC are not fully understood but are associated with multiple factors, such as genetic background, environment, and mucosal immune dysregulation [9]. The interplay of genetic and environmental factors is highly associated with inflammatory bowel disease (IBD), resulting in compromised intestinal barrier function and the activation of the immune response [10]. In this context, one notable pathophysiological characteristic of UC is chronic mucosal inflammation [11]. When the immune system responds to microbes and their mediators, it produces cytokines and chemokines such as TNF, IL-12 and IL-23, which in turn induce the activation and recruitment of adaptive immune cells. These activated immune cells contribute to epithelial cell damage, dysbiosis, and the perpetuation of gut inflammation [10, 12]. Over time, persistent inflammation can cause changes in colon lining cells, eventually increasing the risk of CRC. UC has been demonstrated to be a risk factor for CRC from an inflammatory perspective [13–15].

To efficaciously treat UC and CRC, a recent strategy involved the application of immunomodulatory drugs. Anti-TNF therapies, specifically for UC patients unresponsive to conventional treatments such as corticosteroids, have demonstrated improved clinical outcomes [10]. Furthermore, in the case of CRC, FDA-approved immune checkpoint inhibitors (ICIs) targeting PD-1 and CTLA4 are employed as immunotherapies [16]. Nevertheless, the efficacy of ICI treatment is influenced by the microsatellite instability status (MSI) of each CRC patient [15, 16]. ICI drugs directed at PD-1 and CTLA4 exhibit increased efficacy in metastatic CRC patients with high MSI [17]. Additionally, approximately one-third of UC patients exhibit resistance to anti-TNF therapy [18]. Moreover, as there is no definitive cure for UC thus far, available treatments only help manage and decrease remission periods [19, 20].

To gain a deeper understanding of UC and CRC, both of which are facing such challenges, it is essential to explore the immune environment. Given that both diseases involve immune system remodeling and chronic inflammation, targeting immune modulators is a valuable approach for their treatment. To investigate the alterations in immunological responses, we employed single-cell RNA sequencing (scRNA-seq) profiles of immune cells from colorectal tissues obtained from healthy donors, UC patients, and CRC patients. Our research explored the composition of immune compartments and revealed promising therapeutic targets for UC and CRC based on cellular interactions.

## Materials and methods

### Selection criteria for the public scRNA-seq dataset

To compare the immunological function of immune cells at single-cell resolution, we analyzed scRNA-seq data of colorectum-resident immune cells. The GSE125527 dataset from the Gene Expression Omnibus (GEO) and the E-MTAB-8107 dataset from the European Bioinformatics Institute (EMBL-EBI) database were used [21, 22]. To minimize the influence of technical factors, we selected datasets whose scRNA-seq libraries were generated by 10x Genomics, aligned to the human reference genome hg38, and processed to create a UMI count matrix through 10x Genomics Cell Ranger software. The GSE125527 dataset supplies counts on CD45+ immune cells from intestinal biopsies, while the E-MTAB-8107 dataset provides transcripts on CRC neoplasm core tissue, including immune cells and nonimmune cells. Furthermore, seven UC patients from GSE125527 received medical therapy, 4 of whom received 5-aminosalicylates, 1 of which received azathioprine, 1 of which received vedolizumab, and 1 of which received tofacitinib. Conversely, patients with CRC in the E-MTAB-8107 cohort had undergone surgery without prior treatment.

### Processing of the scRNA-seq dataset

We clustered cells by performing Louvain’s algorithm and confirmed that GSE125527 was composed of immune cells (S1A and S1B Fig) whereas E-MTAB-8107 consisted of seven immune cell populations numbered 1, 2, 5, 6, 7, 11, and 12 among the thirteen clusters (S1C and S1D Fig). Thus, we selected and included only immune cell clusters. After the data were collected, we used the ‘Seurat’ (version 4.0.4) package in the R program (4.1.1) for quality control, dataset integration, normalization, and clustering [23]. We filtered out the cells with a mitochondrial RNA percentage of more than 10%. The data were normalized using NormalizaData, and highly variable features were recognized through the FindVariableFeatures function. Then, sets of anchors between each subject and integration were completed using FindIntegrationAnchors and IntegrateData. Samples with fewer than 500 cells were removed, and we finally obtained a total of 19 patients, 7 healthy donors, 7 UCs, and 4 CRC patients.

### Integration and cell type identification

During the process of integrating the entire dataset, the component of each group with the most number of cells was used as a reference. We then normalized the combined data using SCTransform. The first 50 PCs, which were determined by elbow plots, were utilized for scaling and PCA reduction. Subsequently, we visualized uniform manifold approximation and projection (UMAP) based on the first 50 dimensions of the data reduction method called ‘harmony’ [24]. When setting the optimal number of clusters, the number of clusters containing 100 or more cells for each state was selected, and nine clusters were suitable. Initial clusters were classified as T cells, B cells, plasma cells, and myeloid cells depending on cell type probability calculated by package named ‘SingleR’ and mean expressions of canonical marker genes [21, 22, 25–28]; T-cell (*CD3D*, *CD3E*, *CD3G*, *CD7*), CD4 T-cell (*CD4*, *TCF7*, *FOXP3*, *IL2RA*), CD8 T-cell (*CD8A*, *CD8B*), NK cells (*NCAM1*, *TBX21*), MAIT cell (*SLC4A10*, *TRAV1-2*, *KLRB1*, *IL7R*, *DPP4*), mast cell (*ENPP3*, *M+S4A2*, *TPSAB1*, *CPA3*), B-cell (*CD19*, *MS4A1*, *CD79A*, *CD79B*), plasma cell (*SDC1*, *SDC1*, *CD27*), macrophage (*CD14*, *FCGR3A*) and myeloid cell (*CD68*, *LYZ*, *AIF1*).

### Differentially correlated gene and functional enrichment analysis

To identify genes differentially correlated with *NCR3+* NK cells compared to *NCR3-* NK cells in the UC group and with *HAVCR2+* NK cells compared to *HAVCR2-* NK cells in the CRC group, we converted cell-level gene expression into pseudobulk gene expression. We employed averaged pseudobulked data and applied the Pearson correlation coefficient using the ddcorAll function inherited from the DCGA package [29]. The correlated gene pairs with p-values greater than 0.05 were filtered out. The genes correlated with *NCR3*+ NK cells from the UC group or with *HAVCR2*+ NK cells from the CRC group were identified based on the criteria of gene pairs that exhibited a positive correlation in *NCR3/HAVCR2*+ NK cells but not in *NCR3/HAVCR2*- NK cells.

To explore the functional characteristics of the identified correlated gene sets, we initially conducted a coexpression module analysis using the CEMiTool [30]. Subsequently, we carried out an overrepresentation analysis of the genes in each module utilizing the Gene Annotation & Analysis Resource Metascape [31]. We set a cutoff p-value of 0.05, and the immune-related terms were visualized.

### Gene module scores

We evaluated the degree of cytotoxicity and exhaustion by calculating predefined gene expression values for individual cell types. The cell scores were computed using the AddModuleScore function in Seurat with default settings. We used 12 cytotoxicity-associated genes (*PRF1*, *IFNG*, *GNLY*, *NKG7*, *GZMB*, *GZMA*, *GZMH*, *KLRK1*, *KLRB1*, *KLRD1*, *CTSW*, *and CST7*) and 6 well-defined exhaustion-associated markers (*LAG3*, *TIGIT*, *PDCD1*, *CTLA4*, *HAVCR2*, *and TOX*) to calculate cytotoxicity and exhaustion scores [32].

### Cell‒cell communication

Cell‒cell interactions were quantitively inferred based on known ligand–receptor pairs in identified cell types using the ‘CellChat’ (version 1.1.3) package [33]. We loaded transform-based normalized gene expression values as input and ran CellChat on each group. We used a ligand‒receptor interaction database for humans and followed the official workflow. We identified overexpressed ligand–receptor pairs and projected gene expression data onto the protein–protein interaction network using identifyOverExpressedGenes, identifyOverExpressedInteractions and projectData. The communication probability between cell types was computed by ComputeCommunProb. Finally, the respective estimated cell‒cell interactions for each group were merged. Communication was visualized through built-in graphic functions and the ‘ggplot2’ package [34].

### Gene set enrichment/variation analysis (GSVA)

To explore the pathway enrichment score in NK cells, we conducted GSVA using the R package scGSVA (v0.0.14) [35]. An annotation database was built with biological functions in the Gene Ontology (GO), and the normalized enrichment score (NES) for each cell was calculated. A heatmap was generated from the scGSVA data using the heatmap function.

### Human specimen collection

Colon and rectum specimens from UC patients and healthy donors were procured through the Korea Biobank Network. UC tissues were acquired via diagnostic endoscopic biopsies, encompassing 5 patients from the colon and 6 patients from the rectum. Additionally, 10 normal colon tissues and 10 rectal tissues were collected during colonoscopies for health screenings. The demographic details of the subjects are presented in S1 Table.

### Ethics approval and consent to participate

This procurement was conducted with the approval of the Ethics Committee at Gachon University Gil Medical Center (GBIRB2022-138). Informed consent was duly obtained from all participants. The data were collected for research purposes after September 29, 2022, and only accessed personal information provided by the Korea Biobank Network.

### Immunostaining

Paraffinized tumor tissue blocks were processed into sections and deparaffinized, followed by incubation with 3% hydrogen peroxide to block endogenous peroxidases. The tissue sections were blocked with 1% bovine serum albumin and then incubated with polyclonal antibodies against BAG-6 (Abcam, Cat.No. ab137076), CLEC10A (OriGene, Cat.No. TA810180), KLRD1 (AbboMax, Cat.No. 605–080) and NCR3 (LSBio, Cat.No. LS-C334983-20) overnight at 4°C. The tissues were then stained with goat anti-rabbit IgG H&L (Alexa Fluor® 488 or Alexa Fluor® 594). Confocal images were analyzed using a Carl Zeiss CLSM21 laser scanning microscope and the ZenBlue program (magnification: ×200).

### Survival analysis of COAD dataset from The Cancer Genome Atlas (TCGA)

To validate our findings in CRC, we obtained transcriptional profile data from TCGA under the COAD project. Patients were stratified based on HAVCR2 expression levels using the surv_cutpoint function from the survminer R package. Kaplan-Meier survival analysis and log-rank test were conducted to compare the groups.

## Results

### A scRNA-seq profile of immune cells from healthy controls, UC patients, and CRC patients

**Fig 1:**
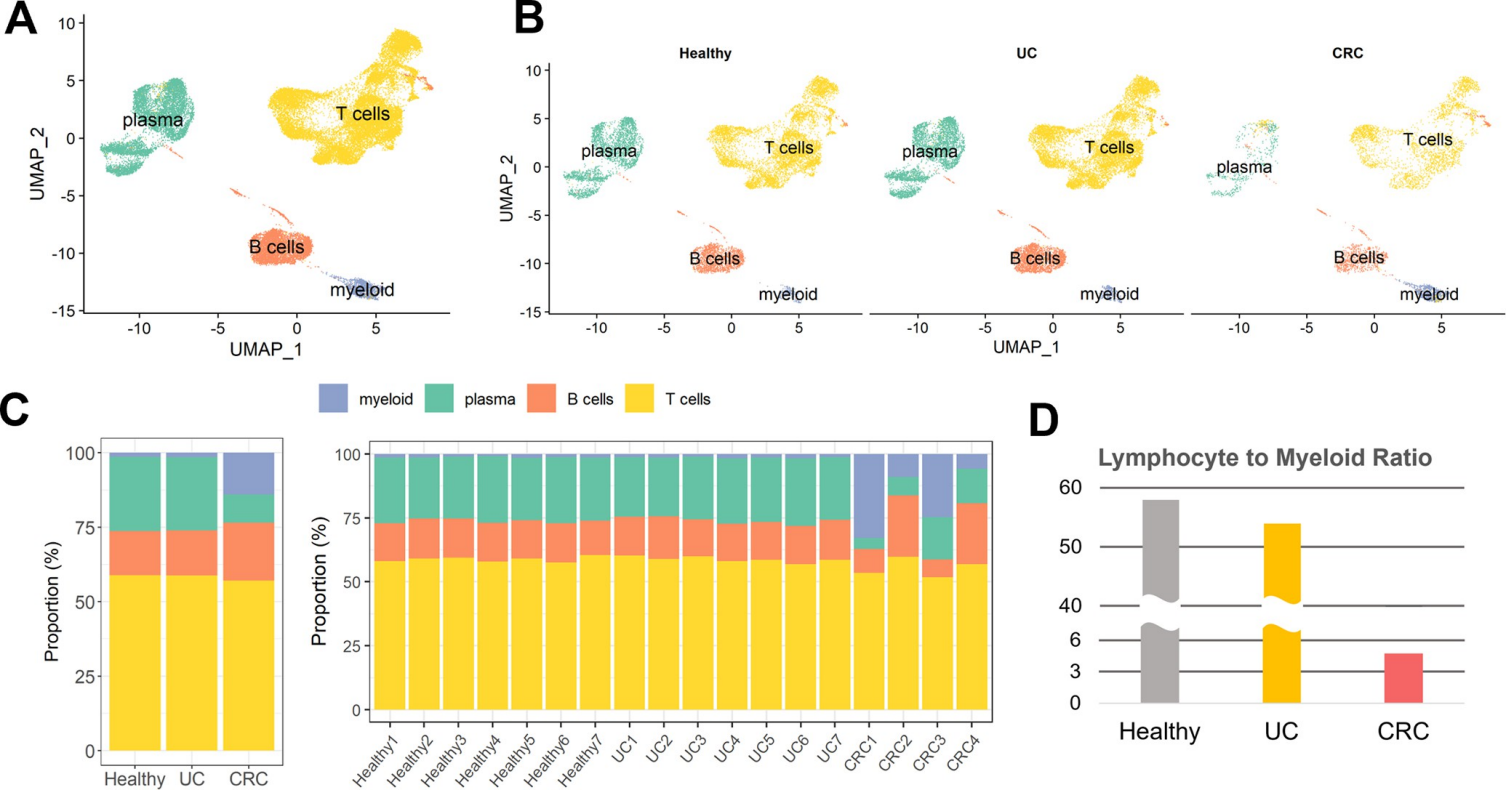
Overview of the scRNA-seq profiles of immune cells from healthy controls, UC, and CRC patients. (A) UMAP plot of annotated cell types. Myeloid, T, B, and plasma cells were identified. (B) UMAP visualization split by disease status. (C) Stacked bar plot of immune cell proportions. The proportions for each group are shown in the left panel, and those for each individual are shown in the right panel. (D) Lymphocyte-to-myeloid ratio according to disease status.

We hypothesized that the immune system is remodeled by status in healthy controls, UC patients, and CRC patients. Thus, we extracted immune cells by identifying the populations. In total, 34,209 immune cells were included and integrated; these cells consisted of 12,193 cells from seven healthy donors, 16,909 cells from seven UC patients, and 5,107 cells from four CRC patients. The integrated data exhibited no obvious batch effects, indicating that sample sequencing techniques had less influence on the data (S2A Fig). These immune cells were segregated into nine populations and identified based on the expression of canonical marker genes (S2B and S2C Fig). Our initial rough clustering identified the cells as T cells, B cells, myeloid cells or plasma cells (Fig 1A). In addition, the cells were uniformly distributed across health conditions (Fig 1B). We subsequently investigated the cellular composition fraction (Fig 1C). There were no notable differences in cellular compartments between healthy donors and UC patients. However, compared with those in the other groups, CRC patients in the other groups had greater proportions of myeloid cells and a lower ratio of plasma cells, although there were some variations, to some extent, depending on the individual. Additionally, the lymphocyte-to-myeloid cell ratio, which is a negative indicator of inflammation and poor prognosis, exhibited a steep decrease in CRC patients (Fig 1D). These findings imply that CRC substantially alters the cellular composition of treated UC patients, whereas the cellular composition of treated UC patients is similar to that of healthy individuals.

### The compositional alteration of immune cell subtypes depending on disease status

**Fig 2:**
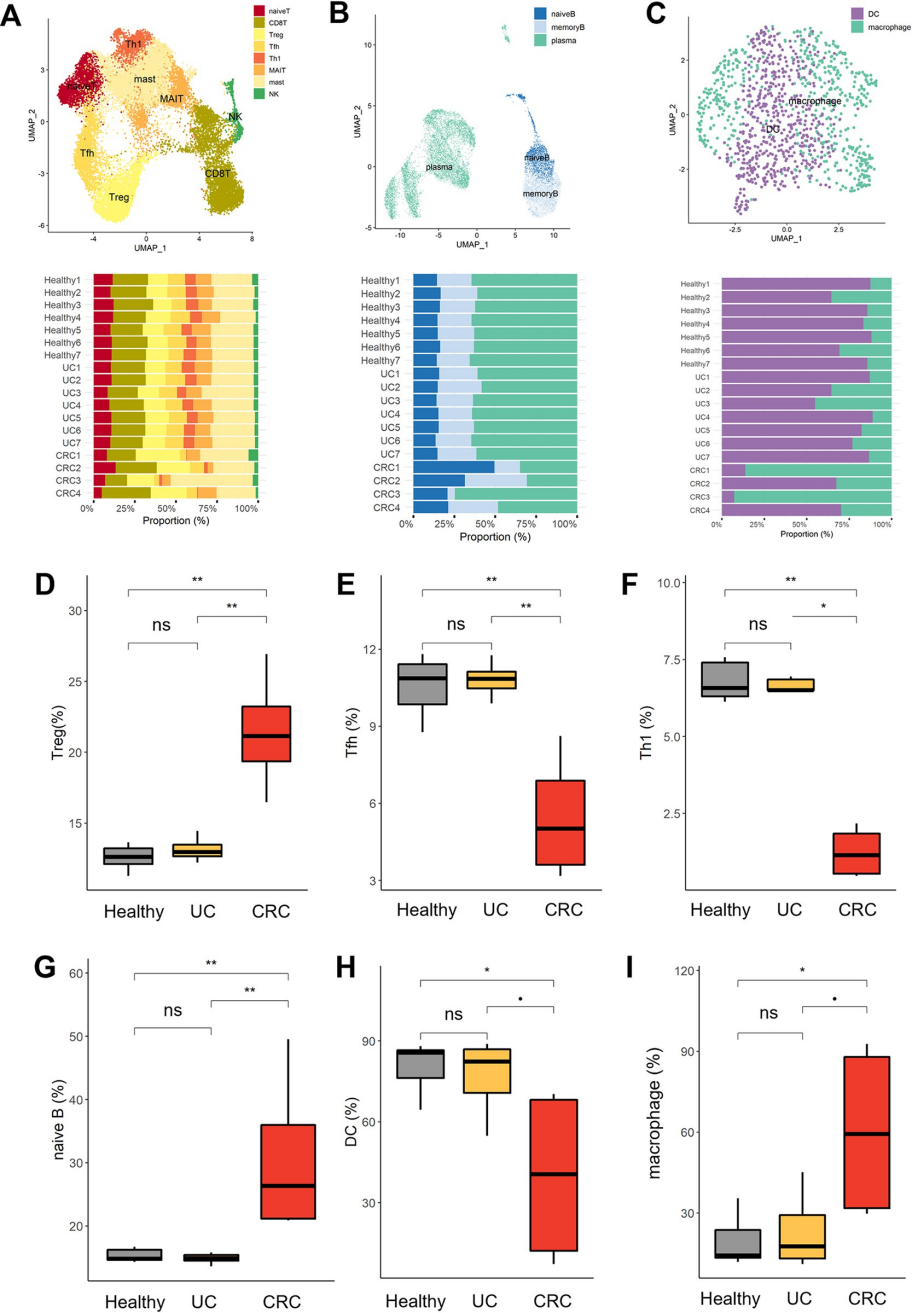
High-resolution cell compartment analysis. (A) UMAP and proportion bar plot of subclustered T cells. The T cells in Fig 1A were reclustered and identified into hierarchical subclusters of T cells and NK cells. The subclusters identified included naive T cells, CD8T cells, regulatory T (Treg) cells, follicular helper T (Tfh) cells, type 1 helper T (Th1) cells, Mucosal-associated invariant T (MAIT) cells, and mast and natural killer (NK) cells. The stacked bar plot shows the proportions of cells in the T-cell lineage and NK cells. (B) UMAP and proportion bar plot of subclustered B cells. As B cells and plasma cells are positioned on the same differentiation lineage, B cells and plasma cells were grouped simultaneously. Subclustering identified naive B, memory B, and plasma cells. The stacked bar plot shows the proportions of B-cell subtypes. (C) UMAP and proportion bar plot of myeloid cells. The myeloid cell group shown in Fig 1A was reclustered and identified as macrophages and dendritic cells (DCs). The stacked bar plot shows the proportions of myeloid cell subtypes. (D-I) The proportions of immune cells in healthy, UC and CRC patients. The boxplot represents the ratio of cellular components for (D) Tregs, (E) Tfh cells, (F) Th1 cells, (G) naive B cells, (H) DCs and (I) macrophages under each condition. The horizontal line indicates the mean ratio, and the Wilcoxon test was performed to compare two groups. p ≤ 0.1 (•), p ≤ 0.05 (*), p ≤ 0.01 (**), p ≤ 0.001 (***), p ≤ 0.0001 (****), not significant (ns).

To determine the fine spectrum of immune cell heterogeneity and states, we reclustered immune cells and identified distinct populations. T lineage cells were identified as naive T, CD8T, Treg, Tfh, Th1, MAIT, mast and NK cells (Fig 2A and S3A Fig), and the B cells were separated into naive B cells, memory B cells, and plasma cells (Fig 2B and S3B Fig). For myeloid cells, DCs and macrophages were revealed (Fig 2C and S3C Fig). Next, we investigated the compositions of T lineages in healthy donors, UC patients, and CRC patients. Compared with healthy donors and UC patients, CRC patients had the largest proportion of regulatory T cells (Tregs) but the lowest proportion of follicular helper T (Tfh) and type 1 helper T (Th1) cells (Fig 2D–2F). Among the B-cell subtypes, UC patients had immunological profiles comparable to those of healthy donors, whereas CRC patients had a greater proportion of naive B cells (Fig 2G). Among myeloid cells, DCs were more common in healthy individuals and UC patients than in macrophages (Fig 2H and 2I). In contrast, although macrophage fractions are considerably increased in certain CRC patients, they exhibit heterogeneous fluctuations according to individual differences. These results suggest that the CRC environment is primarily remodeled by T cells and exhibits heterogeneous perturbations in B and myeloid cells, whereas the UC environment after treatment demonstrates a compositional predisposition parallel to that of normal conditions.

### Comparison of effector cell characteristics between UC patients and CRC patients

**Fig 3:**
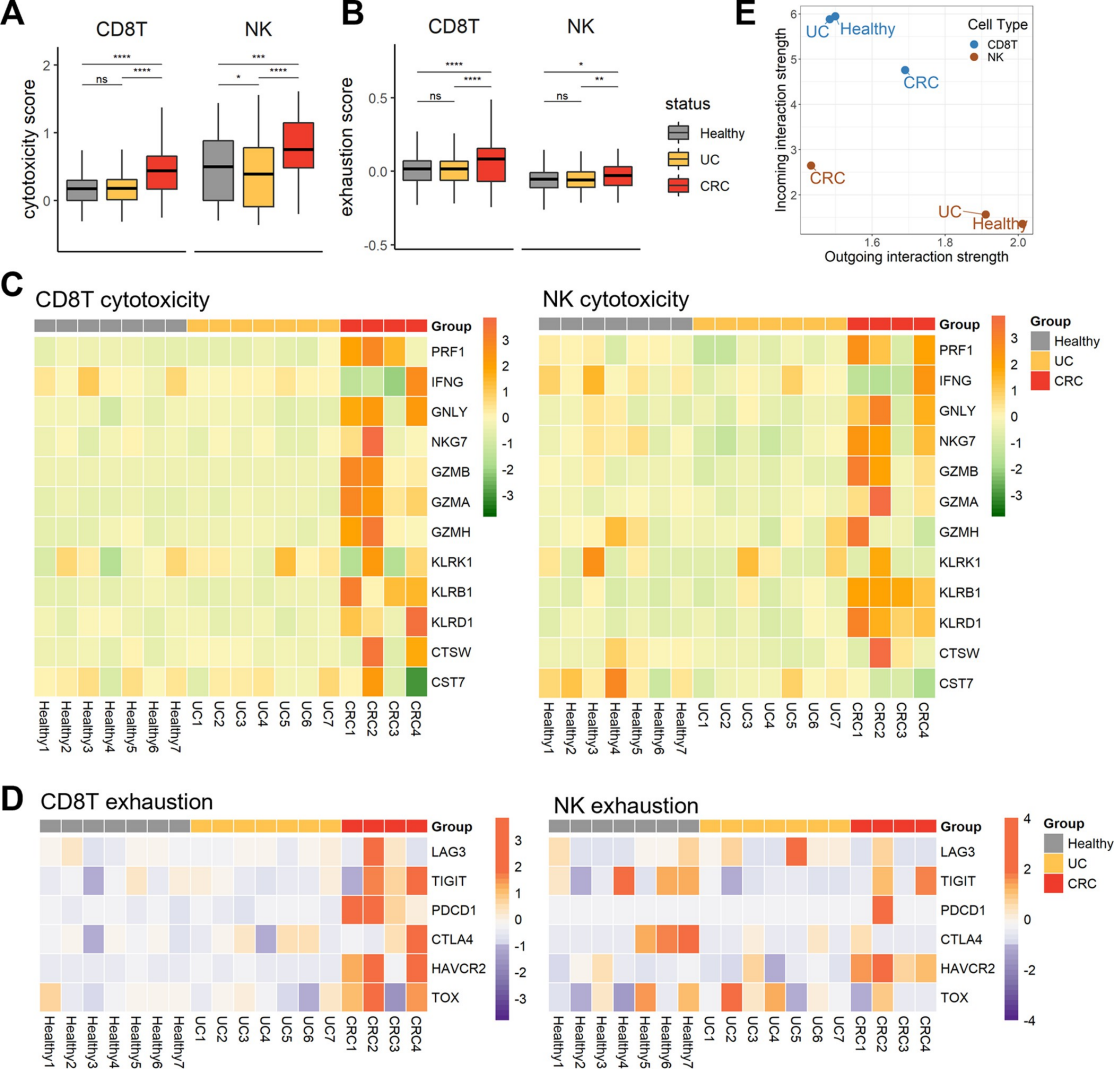
**The altered traits of effector cells, CD8+ T cells, and NK cells.** (A) Box plots of the cytotoxicity scores of NK and CD8+ T cells. The x-axis indicates three groups, healthy, UC, and CRC, and the horizontal line in the box shows the average score. To explore the significance of the differences, the Wilcoxon test was performed; p ≤ 0.05 (*), p ≤ 0.01 (**), p ≤ 0.001 (***), p ≤ 0.0001 (****), and not significant (ns). (B) Box plots of exhaustion scores in NK and CD8+ T cells. Like in Fig 3A, the scores were compared. (C) Heatmap of cytotoxicity-associated genes across participants. The genes used for cytotoxicity scoring are listed, and scaled expression levels of the genes are shown. (D) Heatmap of exhaustion-associated genes spanning participants. The genes used for exhaustion scoring are listed, and scaled expression levels of the genes are shown. (E) Inferred interaction strength of CD8+ T cells and NK cells as a sender and a receiver. The X-axis indicates the outgoing interaction strength of CD8+ T cells and NK cells, which served as signal senders for each condition. The Y-axis indicates the incoming interaction strength of CD8+ T cells and NK cells, which serve as signal receivers for each condition.

While CD8+ T cells and NK cells can kill infected or abnormal cells directly and defend against pathogens, long-lasting secretion of cytotoxic agents can impair tissue cells and lead to chronic inflammation [36]. Therefore, we next explored the attributes of cytotoxic cells, CD8+ T cells and NK cells. We calculated cytotoxicity and exhaustion scores for 12 cytotoxicity-associated genes (*PRF1*, *IFNG*, *GNLY*, *NKG7*, *GZMB*, *GZMA*, *GZMH*, *KLRK1*, *KLRB1*, *KLRD1*, *CTSW*, *and CST7*) and 6 exhaustion-associated genes (*LAG3*, *TIGIT*, *PDCD1*, *CTLA4*, *HAVCR2*, *and TOX*) (Fig 3A). There was a notable alteration in effector function in both UC and CRC patients. In the UC group, NK cells exhibited lower cytotoxicity than the healthy group, although the CD8+ T cells showed no changes in cytotoxicity or exhaustion. In the CRC group, all cytotoxic cells exhibited elevated toxicity, but they also demonstrated a greater level of exhaustion (Fig 3B). CRC patients exhibited increased expression of most cytotoxic genes in both CD8+ T and NK cells (Fig 3C). The CRC group presented heightened levels of granzymes, including *GZMB* and *GZMA*, in CD8+ T cells and *KLRB1* and *KLRD1* in NK cells. Regarding exhaustion, all the CRC patients exhibited increased expression of many inhibitory receptors on CD8+ T cells, especially *PDCD1* and HAVCR2 on NK cells (Fig 3D). To consider the potential for functional changes to be induced by signals exchanged with other cells, we subsequently examined the strength of the cell‒cell interactions between NK and CD8+ T cells (Fig 3E). CD8+ T cells and NK cells were distinctly segregated by the intensity of the received signal rather than the sending signal. CD8+ T cells displayed a sequentially lower intensity of received signals in UC and CRC patients. In contrast, the number of NK cells increased in UC and CRC patients. This observation could imply that NK cells contribute to the reconstruction of the immune microenvironment in both UC and CRC through cytotoxicity and communication with other immune cells.

### UC-specific immune cell interaction network

**Fig 4:**
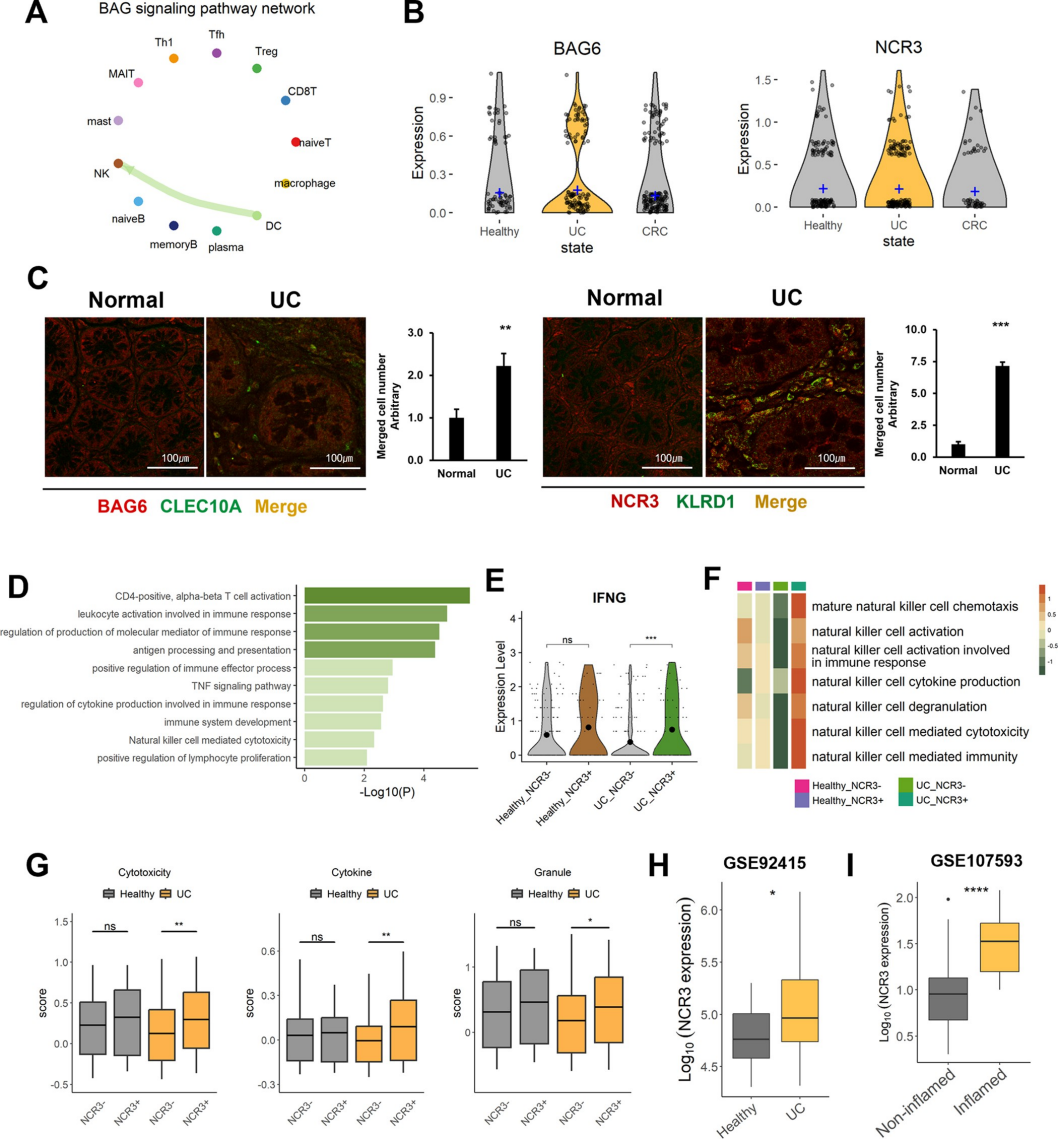
UC-specific cell‒cell communication network among tissue-resident immune cells. (A) Circle plot of cell‒cell interactions in the BAG pathway in UC patients. For each immune cell type, the color of a dot is assigned, and the color of the arrow is the same as the color of the cell type that sends the signal. (B) Gene expression of the ligand *BAG6* and the receptor *NCR3*. The violin plots for *BAG6* are normalized transcripts in DCs (left), and those for *NCR3* are normalized in NK cells (right). The crossbar of the plot indicates the average expression level of each transcript. (C) Comparison of *BAG6* and *NCR3* expression in normal and UC patient tissues through immunostaining. *BAG6* expression was explored in DC (*CLEC10A*) cells, and NCR3 was explored in NK (*KLRD1*) cells. To explore the significance of the difference, the Wilcoxon test was performed; p ≤ 0.05 (*), p ≤ 0.01 (**), p ≤ 0.001 (***), p ≤ 0.0001 (****), and not significant (ns). (D) Functional analysis of differentially coexpressed genes in *NCR3+* NK cells compared to *NCR3-* NK cells in the UC group. Immune-related pathways associated with P<0.05 are shown. (E) Violin plot of *IFNG* expression in healthy donors and UC patients separated by *NCR3* expression in NK cells. The statistical significance was determined using the Wilcoxon test; p ≤ 0.05 (*), p ≤ 0.01 (**), p ≤ 0.001 (***), p ≤ 0.0001 (****), and not significant (ns). (F) GSVA of *NCR3*+ NK cells from UC patients. Heatmaps depicting NK cell-related pathway activities according to the presence of *NCR3* expression in the healthy and UC groups. The color bar indicates the NES, with higher activity represented in orange and lower activity in green. (G) Boxplot of cytotoxicity in NK cells separated according to the expression of NCR3. The groups were separated by the presence (+) or absence (-) of *NCR3* expression in healthy controls and UC patients, and the color is presented according to the disease condition. To calculate the score, we utilized the expression levels of cytokine-encoding genes (*IFNG*, *TNF*, *CSF2*, *IL10*, and *IL15*) and granule-encoding genes (*PRF1*, *GZMB*, *GZMA*, *GZMH*, *GZMM*, *GZMK*, *NKG7*, and *GNLY*). In each panel of the boxplot, the overall cytotoxicity of NK cells was measured using total cytokine- and granule-encoding genes (left), cytokine-mediated toxicity (middle), and granule-mediated toxicity (right). The horizontal line in the box indicates the average score, and Wilcoxon signed-rank test was performed; p ≤ 0.05 (*), p ≤ 0.01 (**), p ≤ 0.001 (***), p ≤ 0.0001 (****), not significant (ns). (H-I) Expression level of NCR3 in GEO datasets, GSE92415 (H) and GSE107593 (I).

To inspect the UC- and CRC-intensified pathways, we investigated ligand and receptor interactions using CellChat. A BAG signal was exclusively detected in UC patients (S4 Fig). Interestingly, the BAG signaling network was composed of the ligand *BAG6* and the receptor *NCR3* (Fig 4A). The signal was sent from the DCs and transferred to the NK cells. In addition, the expression level of *BAG6* in DCs was slightly increased in UC patients (Fig 4B). To examine whether the BAG signal is specifically related to UC status, we conducted immunostaining on tissues obtained from healthy donors and UC patients (Fig 4C). Notably, both *BAG6* in DCs and *NCR3* in NK cells were strongly detected in UC tissues. In contrast, they were rarely found in normal tissues. The level of *NCR3* detected in NK cells from UC patients was approximately 7 times greater than that in NK cells from healthy individuals, consistent with the inferred signaling network. To determine the immunological attributes of *NCR3*+ NK cells in comparison to *NCR3*- NK cells, we identified a coexpression module using genes that are differentially correlated with *NCR3*+ NK cells from CRC patients, and we investigated the biological roles of these genes (Fig 4D). The *NCR3*+ NK cells from UC patients exhibited upregulated expression of genes associated with lymphocyte activation, the TNF signaling pathway, and NK cell cytotoxicity. Additionally, only under UC conditions did NCR3+ NK cells express a greater amount of IFNG, which is known to promote the accumulation and activation of NK cells, than NCR3- NK cells (Fig 4E) [37]. GSVA revealed that NK cell activation and cytokine production exhibited distinctly high activity depending on the presence of NCR3 under UC conditions (Fig 4F). We thus inspected the cytotoxicity of NK cells based on *NCR3* expression by further classifying them into cytokine-mediated and granule-mediated cytotoxic effects (Fig 4G) [38]. Consequently, in the normal state, NK cells exhibit no difference in effector function depending on *NCR3* expression. However, in UC patients, we observed significant differences in the cytotoxicity of NK cells, both in cytokine-mediated and granule-mediated cytotoxicity; the cytotoxicity was greater in the *NCR3+* NK group than in the *NCR3-* NK group. Next, we investigated the expression level of *NCR3* in two GEO datasets, GSE92415 and GSE107593 (Fig 4H and 4I). GSE92415 includes samples from 21 healthy individuals and 87 UC patients, while GSE107593 contains 24 non-inflamed and 24 inflamed tissues obtained from UC patients. Compared to the healthy group and non-inflamed colon tissues, both the UC group and inflamed colon tissues exhibited higher levels of *NCR3*. Collectively, This finding suggested that the NCR3 receptor of NK cells, which receives signals from DCs through BAG signaling, mediates the secretion of cytotoxic molecules and contributes to the persistence of colitis.

### The GALETIN pathway in CRC patients

**Fig 5:**
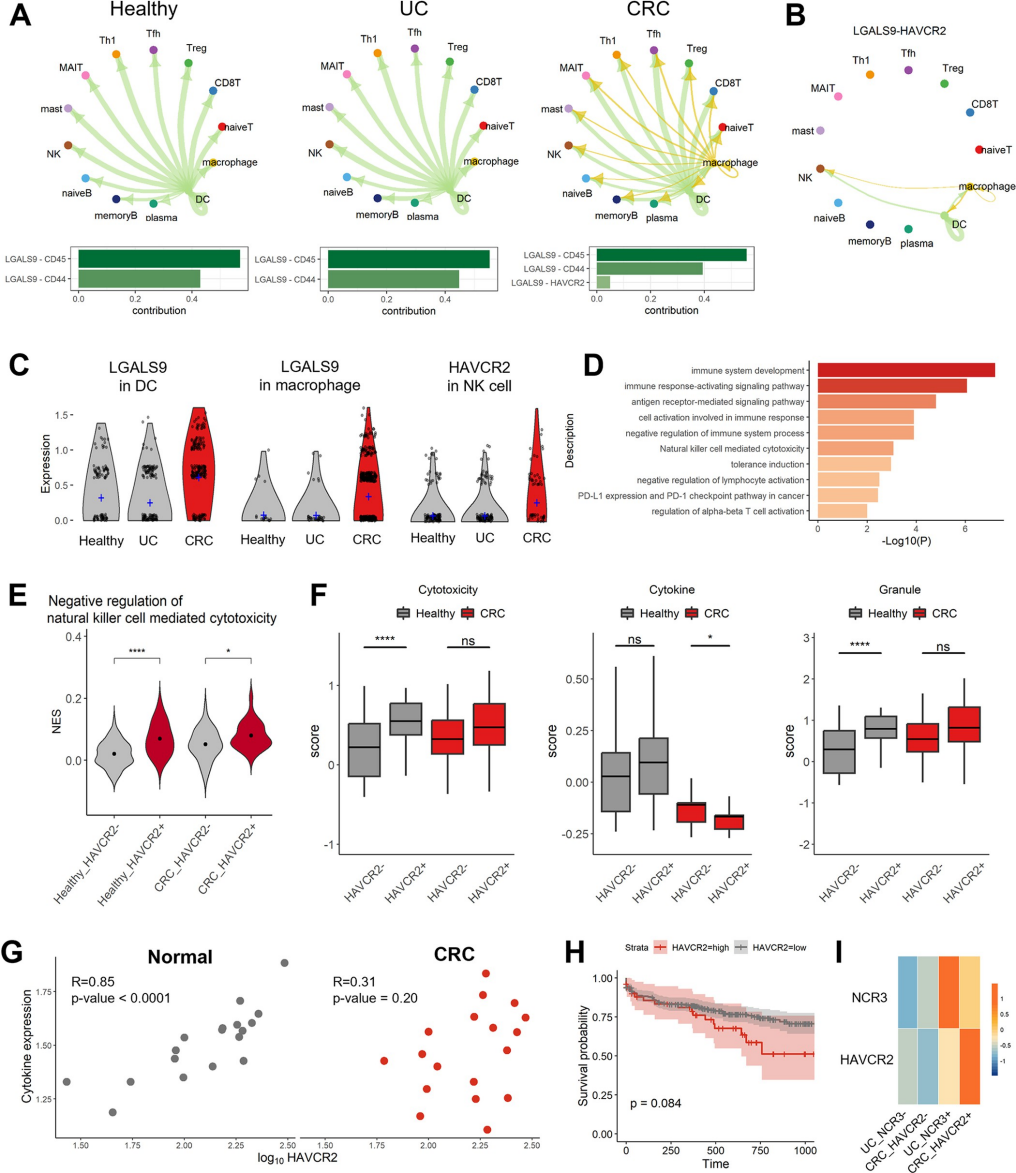
CRC-specific intercellular signaling pathways. (A) The GALECTIN pathway according to disease condition. Each immune cell type is assigned the color of a circle, and the color of the arrow is the same as the color of the cell type sending the signal. (B) Circle plot of the interaction between the *LGALS9*-*HAVCR2* pair and the GALECTIN pathway in healthy donors, UC patients, and CRC patients. (C) Gene expression levels of a ligand and a receptor in the GALECTIN pathway. The violin plot shows the expression levels of the ligand *LGALS9* in DCs and macrophages and the receptor *HAVCR2* in NK cells. The crossbar indicates the average expression level of transcripts in each status. (D) Immunological functions of differentially coexpressed genes in *HAVCR2*+ NK cells compared to *HAVCR2-* NK cells in the CRC group. To determine the significance of the differences, the Wilcoxon test was performed; p ≤ 0.05 (*), p ≤ 0.01 (**), p ≤ 0.001 (***), p ≤ 0.0001 (****), and not significant (ns). (E) Violin plot of NES for negative regulation of natural killer cell-mediated cytotoxicity. The groups were separated based on disease condition and the expression of *HAVCR2*. (F) Boxplot of cytotoxicity in NK cells separated according to *HAVCR2* expression. As shown in Fig 4G, groups were separated by the presence (+) or absence (-) of *HAVCR2* expression in healthy individuals and CRC patients. (G) Correlation between HAVCR2 and cytokine expression levels. The correlation coefficients and p-values are displayed in the upper left. (H) Kaplan-Meier survival plot for 3 years. The 371 CRC patients from TCGA-COAD are classified based on the expression levels of HAVCR2, and survival plot is described in days. (I) The expression level of NCR3 and HAVCR2 in NK cells of UC and CRC patients. The groups are categorized based on the expression levels of NCR3 and HAVCR2 with disease.

We next detected an increase in the GALECTIN signaling pathway within the CRC environment. Among the GALECTIN family members, *LGALS9* (*GALECTIN-9*) plays a primary role in this signaling pathway (Fig 5A). *CD45* and *CD44* were observed as receptors, and DCs sent signals to overall immune cells. However, the *LGALS9*-*HAVCR2* pair was also newly discovered in CRC macrophages and was an additional signal sender. In particular, only NK cells communicated with myeloid cells via *LGALS9*-*HAVCR2* signaling under CRC conditions (Fig 5B and S2 Table). LGALS9 expression was elevated in DCs and macrophages from the CRC group, and the level of *HAVCR2* in NK cells was increased (Fig 5C). From the coexpression module analysis, two modules were extracted from the *HAVCR2*+ NK cells of CRC patients, and their biological functions were inspected (Fig 5D and S5A Fig). The functions associated with the regulation of immune system processes were primarily recognized. Among the enriched terms, we detected the PD-1 checkpoint pathway, NK cell-mediated cytotoxicity, and induction of tolerance. The results of GSVA were consistent with the results of the coexpression module analysis; in particular, *HAVCR2*+ NK cells from CRC patients exhibited strong inactivity of NK cell-mediated cytotoxicity (Fig 5E, and S5B Fig). We also examined the molecular cytotoxicity score of NK cells from healthy individuals and CRC patients stratified by *HAVCR2* expression. Although there was no difference in the granular cytotoxicity score, the cytotoxicity score mediated by cytokine secretion was decreased in the *HAVCR2*+ NK cells of CRC patients. Subsequently, we investigated the association between the expression level of *HAVCR2* and cytokine production in the GSE50760 dataset contating 18 healthy individuals and 18 CRC patients. In the healthy control group, an increase in *HAVCR2* expression levels was linearly associated with higher cytokine production, whereas no significant correlation was observed in the CRC group (Fig 5G). In addition, high levels of HAVCR2 demonstrated worse clinical outcomes in the TCGA-COAD cohort (Fig 5H). Finally, we explored the association of NCR3 and HAVCR2 expression with disease status (Fig 5I). Interestingly, the NCR3+ UC group exhibited the second-highest expression level of HAVCR2, following the HAVCR2+ CRC group. In addition, the HAVCR2+ CRC group showed the second-highest level of NCR3, following the NCR3+ UC group. Considering these findings, we suggest that the *LGALS9*/*HAVCR2* crosstalk found only in CRC interferes with the killing function of NK cells through cytokine secretion rather than via granule formation and suggest that *HAVCR2* is an important immunological target for improving cytokine levels in CRC. Furthermore, we observed a potential correlation between NCR3-expressing NK cells in UC patients and HAVCR2-expressing NK cells in CRC patients, indicating a possible shared mechanism or pathway in these distinct conditions.

## Discussion

This research explored the alterations in the immune response observed in UC and CRC, which are increasingly prevalent diseases and originate in the same anatomical region. As a treatment for UC, a step-up therapeutic strategy is employed. As an initial therapeutic strategy, medication is administered to suppress colonic inflammation and promote tissue regeneration [39]. However, depending on the patient’s reaction to drug treatment, this approach can be extended to surgical intervention [40]. In the context of CRC, treatment is determined by the cancer stage. Early-stage CRC might be adequately managed with partial colectomy, whereas advanced-stage CRC necessitates a comprehensive treatment approach, including chemotherapy, radiotherapy, and targeted therapies [41]. Therefore this study elucidated the immune cell characteristics of UC patients who had undergone medication treatment and CRC patients who had not received such interventions.

Previous studies have revealed that in UC patients, CD8+ T cells and NK cells secrete proinflammatory cytokines, such as IL-17, IL-4, IL-5, and IL-13, stimulating leukocyte migration and secretion of subsequent cytokines [42–45]. In the CRC environment, immune cell infiltration, such as DC, cytotoxic T, and NK cells, decreases, and regulatory B cells express PD-L1 leading to exhaustion of T cells [46–49]. This paper focused on the modifications of cytotoxic effector cells, CD8+ T cells and NK cells. Under CRC conditions, there were significant alterations in both NK cells and CD8+ T cells. The exhaustion of both types of cytotoxic cells in CRC patients aligns well with the findings of previous studies [50–52]. In contrast, compared with those in healthy donors, NK cells in UC patients did not exhibit any alterations in exhaustion but displayed decreased cytotoxicity. Reduced cytotoxicity of circulating NK cells was also reported in active UC patients who received medication [53]. Therefore, the medication applied to alleviate the symptoms of UC was generally effective. Nonetheless, the following signaling pathway mediated by NK cells was distinctly detected under UC conditions.

As a UC-specific pathway, the BAG6 signaling pathway was identified. The pathway was organized with the *NCR3* receptor and its ligand *BAG6*. Although the impact of the BAG6 pathway on NK cell cytotoxicity is known to be context dependent, it has been reported that BAG6-NCR3 crosstalk is integral for regulating immune responses, particularly NK cell activity and dendritic cell maturation, in non-small cell lung cancer and chronic lymphocytic leukemia [54–56]. BAG6, a nuclear protein, is released in response to stress signals from tumor and dendritic cells [55]. Subsequently, NK cells are activated through the BAG6-NCR3 axis and subsequently induce the lysis of target cells [57]. Immunostaining of UC colon tissues supported the presence of this pathway. Consistent with the results of previous reports, NK cells mediated by *NCR3* in UC patients presented a higher level of *IFNG*, which is typically expressed upon the activation of NK cells, and the highest cytotoxicity score, whereas there was no difference in healthy individuals. This finding suggested that even following treatment for UC, a residual inflammatory signal was continued through the BAG6 pathway. Unresolved persistent inflammation in UC can lead to fibrosis of the intestinal mucosa and submucosa, causing structural changes in the intestinal tissue. This can interfere with normal cell function, including that of immune cells, thereby increasing the risk of colorectal cancer [58, 59]. Moreover, since traditional therapeutic drugs have limitations, in that they provide only temporary symptom relief and do not alter the long-term disease progression [60], targeting NCR3 in NK cells via the BAG6 pathway could be a promising strategy when combined with current therapies.

HAVCR2, which is also known as TIM3, is a type I transmembrane protein that plays a crucial role in activating and restraining immune responses [61, 62]. HAVCR2 is found in several immune cells, including T cells, DCs, and NK cells [63]. While many studies have focused on the function of HAVCR2 in T cells, we observed an increase in HAVCR2 in NK cells from all CRC patients, and the LGALS9-HAVCR2 axis was found to be specifically involved in NK cell function in CRC. The HAVCR2 gene of NK cells is responsible for the suppression of NK cell-mediated cytotoxicity in peripheral blood mononuclear cells [63]. The ability of NK cells to restrict cytokines in a GALECTIN-9-dependent manner has been reported in response to malignant cells and chronic infection [64, 65]. Additionally, it has been observed that HAVCR2+ NK cells in the periphery are negatively correlated with the clinical stage of CRC [66]. In the same context, HAVCR2+ NK cells from CRC tissue exhibited upregulated expression of genes associated with the immune checkpoint pathway and downregulated NK cell-mediated cytotoxicity. In light of these observations, it becomes evident that HAVCR2+ NK cells potentially contribute to tumor immune evasion. This underscores the need for further research into targeted therapies that can modulate the LGALS9-HAVCR2 axis, offering new avenues for enhancing NK cell cytotoxicity in CRC treatment.

Our findings suggest the potential for establishing therapeutic targets by inhibiting NCR3 in UC and HAVCR2 in CRC patients. Future studies should focus on the development and application of antagonists or blocking antibodies targeting the NCR3 receptor on NK cells in UC patients and the HAVCR2 receptor on NK cells in CRC patients. To validate these potential therapeutic strategies, experiments using in vitro and animal models could be applied. These studies should assess the modulation of inflammatory responses, cell proliferation and invasion, as well as potential off-target effects. Moreover, for patients with UC-associated CRC, it would be valuable to explore the synergistic effects of targeting both NCR3 and HAVCR2 simultaneously. This approach may provide insights into enhanced therapeutic efficacy for UC and CRC treatments.

However, this study has limitations that should be considered. Firstly, further research is needed based on the stratification of pathological factors and treatment regimens. As outcomes vary depending on confounding factors such as the duration of inflammation and disease severity, it is necessary to develop strategies that take these factors into account. Secondly, since this study proposes NCR3 and HAVCR2 receptors on NK cells, as therapeutic targets, additional exploration is needed to generalize these findings. This could be achieved through large-scale single-cell transcriptome cohorts or sorting experiments to identify NK cells. Additionally, it is essential to investigate the roles of NCR3 and HAVCR2 in other immune cells.

In summary, this study investigated the immune microenvironment of UC and CRC patients. We examined immunological changes and NK cell signaling in UC and CRC, revealing treatment limitations and proposing new adjuvant therapies.

## Supporting information

S1 Table Demographics of participants for human specimen collection. (DOCX)

S2 Table Ligand-receptor communication probability of GALECTIN pathway. Cell type in row indicates sender of pathway signal and cell type in column means receiver of signaling. Each sheet represents the detected pair of GALECTING pathway.
(XLSX)

S1 Fig The process to distinguish hematopoietic immune cells from the non-immune cell population. (A) UMAP of public scRNA-seq data from GSE125527. We extracted cells isolated from rectal biopsies in GSE125527 and included them in this study. The cells are clustered and grouped into 11 clusters. (B) Dot plot of immune cell and non-immune cell marker. To verify that all included cells were hematopoietic cells, the expression levels of each group were confirmed using representative immune cells and non-immune cell markers. B cells (*CD79A*, *CD79B*), T cell (*CD3D*, *CD3E*, *CD3G*), DC (*CD1C*, *CD207*, *CLEC9A*, *LILRA4*, *CCL17*), mat cell (*MS4A2*, *TPSAB1*, *CPA3*), myeloid (*CD68*, *LYZ*, *AIF1*), NK (*NCAM1*,*FCGR3A*,*CD7*,*TBX21*), macrophage (*CD14*), cancer cell (*EPCAM*, *KRT7*, *KRT18*), endothelial cell (*CLDN5*,*PECAM1*,*VWF*), enteric glia (*S100B*, *PLP1*), fibroblast (*COL1A*, *BGN*, *DCN*) and epithelial cell (*MT1E*, *MT1G*,*ITLN1*, *ZG16*). Unmarked genes were not included genes in this dataset. (C) UMAP of public scRNA-seq data from E-MTAB-8107. We chose data sampled from the colorectum neoplasm core site of CRC patients. (D) Dot plot of immune cell and non-immune cell marker. The identical markers of S1B Fig were used and non-immune cell clusters are excluded.
(DOCX)

S2 Fig UMAP of the integrated dataset. (A) UMAP visualization splitted by sequencing platform. To minimize the technological batch effect, we select public data which produced scRNA-seq data using an identical platform. Both GSE125527 and E-MTAB-8107 prepared and generated scRNA-seq data using the protocol of 10x Genomics. 10X-1 means the platform of GSE125527 while 10X-2 indicates the platform of E-MTAB-8107. (B) UMAP projection of 32,209 cells. Each dot represents one cell and cells are grouped by transcriptional profile. Nine clusters were identified. (C) Dot plot of the canonical immune cell marker gene. Expression values were represented by the intensity of color and dot size to portray the proportion of cells expressing marker genes in each cluster.
(DOCX)

S3 Fig UAMP and dot plots for identifying sub-clustered cell components. (A) sub-clustered UMAP and dot plot of T cells. T cells in Fig 1B are re-grouped into 12 identities. The cell components were annotated using cell specific markers of dot plot; T cell (*CD3D*, *CD3E*, *CD3G*), naive T cells (*SELL*, *CCR7*, *TCF7*, *LEF1*), CD8T cell (*CD8A*, *CD8B*), CD4T cell (*CD4*), Treg (*FOXP3*, *IL2RA*, *ICOS*), Tfh *(MAF*, *PDCD1*, *CXCR5*), MAIT (*SLC4A10*, *TRAV1-2*, *KLRB1*, *IL7R*, *DPP4*), mast cell (*ENPP3*, *MS4A2*, *TPSAB1*, *CPA3*) and NK (*KLRD1*, *FCGR3A*). We identified naive T (cluster 8,9), CD8 T (cluster 2,5), Treg (cluster 1), Tfh (cluster4), Th1 (cluster 7), MAIT (cluster 6,10), mast cell (cluster 0,3), and NK (cluster 11). (B) sub-clustered UMAP and dot plot of B cells and plasma cells. B cells and plasma cells in Fig 1B are re-grouped into 12 identities. The cell components were annotated using cell-specific markers of dot plot; naive B (*BACH2*, *IGHD*, *FCER2*), memory B (*EBF1*, *MS4A1*, *HLA-DRA*, *PAX5*, *CD22*, *CD19*, *IRF8*, *CD27*), and plasma cell (*IRF4*, *PRDM1*, *SDC1*, *CD38*). We identified naive B (cluster 1,9), memory B (cluster 3,4) and plasma cell (cluster 0,2,5,6,7,8,10,11). (C) sub-clustered UMAP and dot plot of myeloid cells. Myeloid cells in Fig 1B are re-grouped into 9 identities. The cell components were annotated using cell-specific markers of dot plot; DC (*CD1C*, *CD33*, *CLEC10A*, *FCER1A*, *FCGR2B*) and macrophage (*FCGR3A*, *ITGAM*, *FCN1*, *S100A9*, *VCAN*). We identified DC (cluster 1,2,3,7,8) and macrophages (cluster 0,4,5,6).
(DOCX)

S4 Fig Comparison of cell-cell crosstalk between UC and CRC. Relative information flow of significant signaling pathway. The color of the pathway on the y-axis means enriched pathway in the specific condition such as healthy control (grey), UC (yellow), and CRC (red). Pathway in black refers to the equally important pathway in two groups. Bar size implies the relative information flow, which is computed using the communication probability of a particular signaling pathway. The left bar plot compares the signaling pathway of healthy and UC status and the right bar plot compares healthy and CRC status.
(DOCX)

S5 Fig Biological function of *HAVCR2*+ NK cell of CRC patients. (A) Immunological functions of differentially coexpressed genes of the other module detected in *HAVCR2*+ CRC NK cells. (B) GSVA pathway for *HAVCR2*+ NK cell in CRC patients. Heatmaps depicted NK cell-related pathway activities depending on the presence of *HAVCR2* expression in healthy and CRC groups. The colorbar describes the NES, with higher activity represented in orange and lower activity in green.
(DOCX)

S1 Graphical abstract (TIFF)

## Abbreviations

CRC: colorectal cancer
EMBL-EBI: European Bioinformatics Institute
GEO: Gene Expression Omnibus
GO: Gene Ontology
IBD: inflammatory bowel disease
ICI: immune checkpoint inhibitor
MSI: microsatellite instability status
NES: normalized enrichment score
scRNA-seq: single-cell RNA sequencing
Th1: type 1 helper T
Tfh: follicular helper T
Treg: regulatory T cell
UC: ulcerative colitis
UMAP: uniform manifold approximation and projection
UMI: unique molecular identifier
