# Supplementary material for: Uncovering NK cell sabotage in gut diseases via single cell transcriptomics
Source: PLoS One. 2025 Jan 3;20(1):e0315981. doi: 10.1371/journal.pone.0315981 (PMC11698320; doi:10.1371/journal.pone.0315981)
Supplement: S1 Table — (DOCX) [file pone.0315981.s001.docx]

**S1 Table. Demographics of participants for Human specimen collection.**

|  |  | No | Age (Mean) | Male (No) | DM (No) | HTN (No) |
| --- | --- | --- | --- | --- | --- | --- |
| UC | Colon | 5 | 48.2 | 4 | 0 | 0 |
|  | Rectum | 6 | 43.4 | 2 | 0 | 1 |
| Normal | Colon | 10 | 69.9 | 5 | 1 | 3 |
|  | Rectum | 10 | 69 | 6 | 4 | 4 |

HTN: hypertension
