# Supplementary material for: Uncovering NK cell sabotage in gut diseases via single cell transcriptomics
Source: PLoS One. 2025 Jan 3;20(1):e0315981. doi: 10.1371/journal.pone.0315981 (PMC11698320; doi:10.1371/journal.pone.0315981)
Supplement: S1 Fig — (A) UMAP of public scRNA-seq data from GSE125527. We extracted cells isolated from rectal biopsies in GSE125527 and included them in this study. The cells are clustered and grouped into 11 clusters. (B) Dot plot of immune cell and non-immune cell marker. To verify that all included cells were hematopoietic cells, the expression levels of each group were confirmed using representative immune cells and non-immune cell markers. B cells (CD79A, CD79B), T cell (CD3D, CD3E, CD3G), DC (CD1C, CD207, CLEC9A, LILRA4, CCL17), mat cell (MS4A2, TPSAB1, CPA3), myeloid (CD68, LYZ, AIF1), NK (NCAM1,FCGR3A,CD7,TBX21), macrophage (CD14), cancer cell (EPCAM, KRT7, KRT18), endothelial cell (CLDN5,PECAM1,VWF), enteric glia (S100B, PLP1), fibroblast (COL1A, BGN, DCN) and epithelial cell (MT1E, MT1G,ITLN1, ZG16). Unmarked genes were not included genes in this dataset. (C) UMAP of public scRNA-seq data from E-MTAB-8107. We chose data sampled from the colorectum neoplasm core site of CRC patients. (D) Dot plot of immune cell and non-immune cell marker. The identical markers of S1B Fig were used and non-immune cell clusters are excluded. (DOCX) [file pone.0315981.s003.docx]

**S1 Fig. The process to distinguish hematopoietic immune cells from the non-immune cell population.**

**
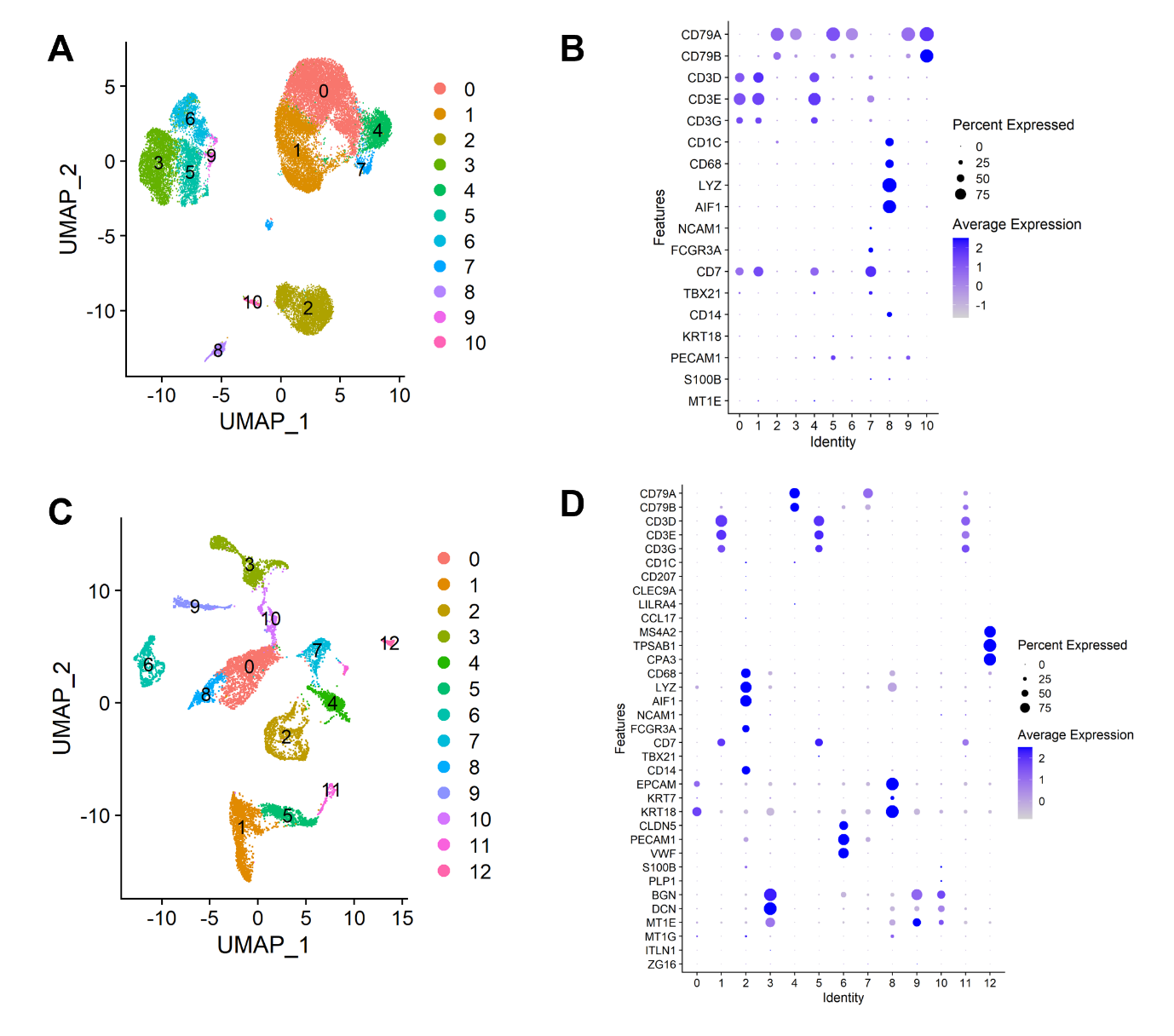
**
