# Supplementary figures and images for: Uncovering NK cell sabotage in gut diseases via single cell transcriptomics
Source: PLoS One. 2025 Jan 3;20(1):e0315981. doi: 10.1371/journal.pone.0315981 (PMC11698320; doi:10.1371/journal.pone.0315981)

**S2 Fig. UMAP of the integrated dataset.**

**
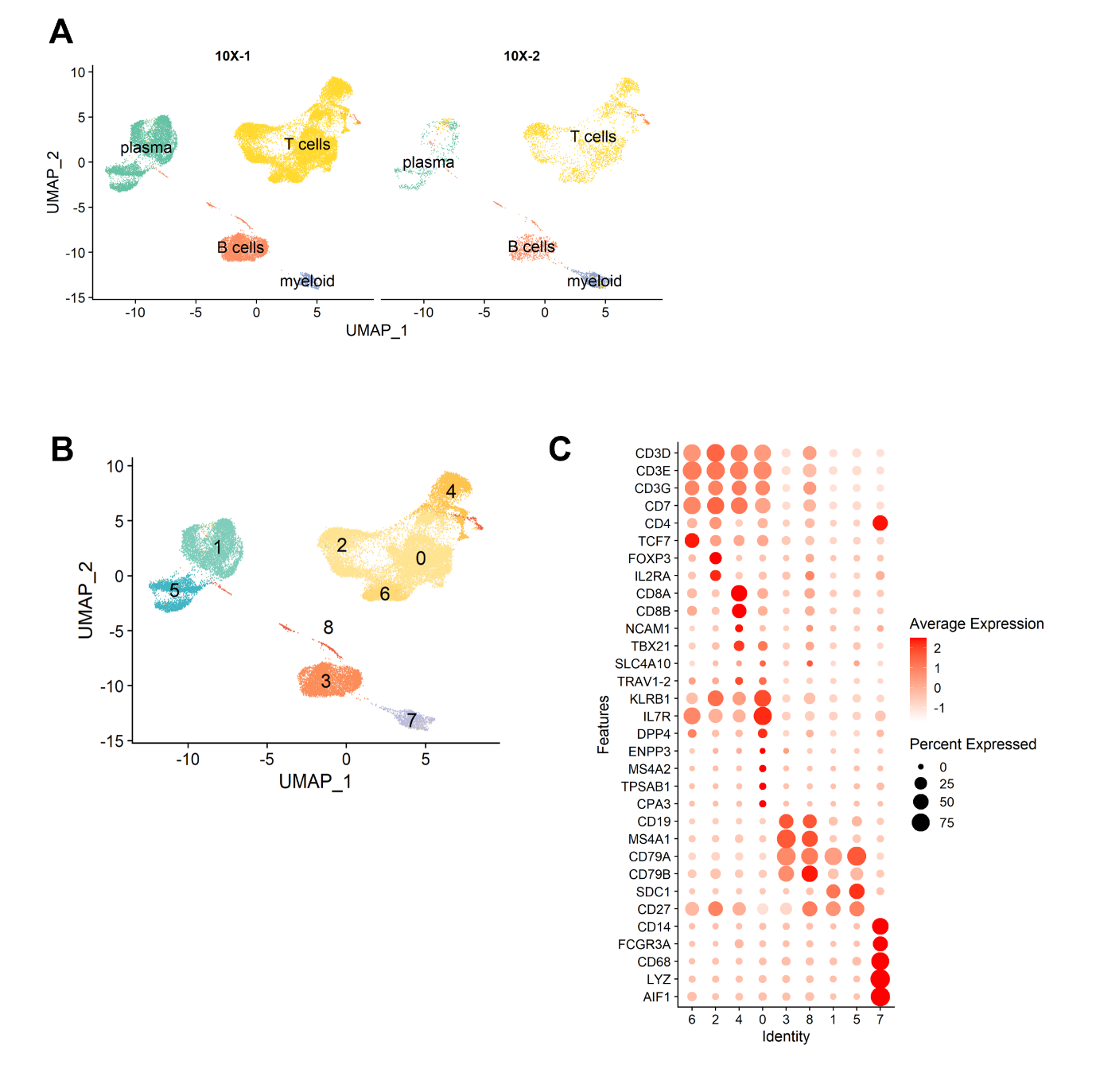
**

Supplement: S2 Fig — (A) UMAP visualization splitted by sequencing platform. To minimize the technological batch effect, we select public data which produced scRNA-seq data using an identical platform. Both GSE125527 and E-MTAB-8107 prepared and generated scRNA-seq data using the protocol of 10x Genomics. 10X-1 means the platform of GSE125527 while 10X-2 indicates the platform of E-MTAB-8107. (B) UMAP projection of 32,209 cells. Each dot represents one cell and cells are grouped by transcriptional profile. Nine clusters were identified. (C) Dot plot of the canonical immune cell marker gene. Expression values were represented by the intensity of color and dot size to portray the proportion of cells expressing marker genes in each cluster. (DOCX) [file pone.0315981.s004.docx]

**S3 Fig. UAMP and dot plots for identifying sub-clustered cell components.**

**
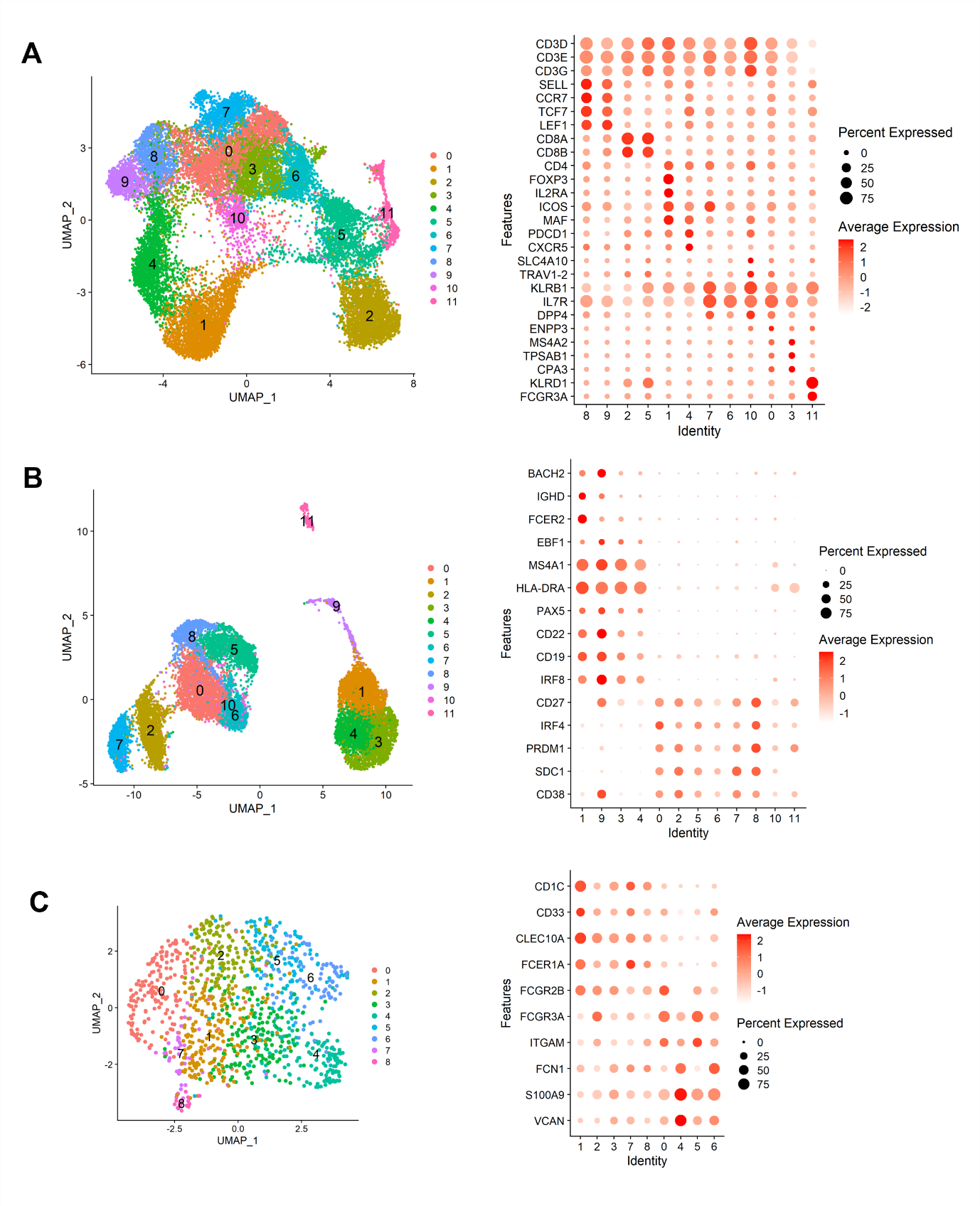
**

Supplement: S3 Fig — (A) sub-clustered UMAP and dot plot of T cells. T cells in Fig 1B are re-grouped into 12 identities. The cell components were annotated using cell specific markers of dot plot; T cell (CD3D, CD3E, CD3G), naive T cells (SELL, CCR7, TCF7, LEF1), CD8T cell (CD8A, CD8B), CD4T cell (CD4), Treg (FOXP3, IL2RA, ICOS), Tfh (MAF, PDCD1, CXCR5), MAIT (SLC4A10, TRAV1-2, KLRB1, IL7R, DPP4), mast cell (ENPP3, MS4A2, TPSAB1, CPA3) and NK (KLRD1, FCGR3A). We identified naive T (cluster 8,9), CD8 T (cluster 2,5), Treg (cluster 1), Tfh (cluster4), Th1 (cluster 7), MAIT (cluster 6,10), mast cell (cluster 0,3), and NK (cluster 11). (B) sub-clustered UMAP and dot plot of B cells and plasma cells. B cells and plasma cells in Fig 1B are re-grouped into 12 identities. The cell components were annotated using cell-specific markers of dot plot; naive B (BACH2, IGHD, FCER2), memory B (EBF1, MS4A1, HLA-DRA, PAX5, CD22, CD19, IRF8, CD27), and plasma cell (IRF4, PRDM1, SDC1, CD38). We identified naive B (cluster 1,9), memory B (cluster 3,4) and plasma cell (cluster 0,2,5,6,7,8,10,11). (C) sub-clustered UMAP and dot plot of myeloid cells. Myeloid cells in Fig 1B are re-grouped into 9 identities. The cell components were annotated using cell-specific markers of dot plot; DC (CD1C, CD33, CLEC10A, FCER1A, FCGR2B) and macrophage (FCGR3A, ITGAM, FCN1, S100A9, VCAN). We identified DC (cluster 1,2,3,7,8) and macrophages (cluster 0,4,5,6). (DOCX) [file pone.0315981.s005.docx]

**S4 Fig. Comparison of cell-cell crosstalk between UC and CRC**

**
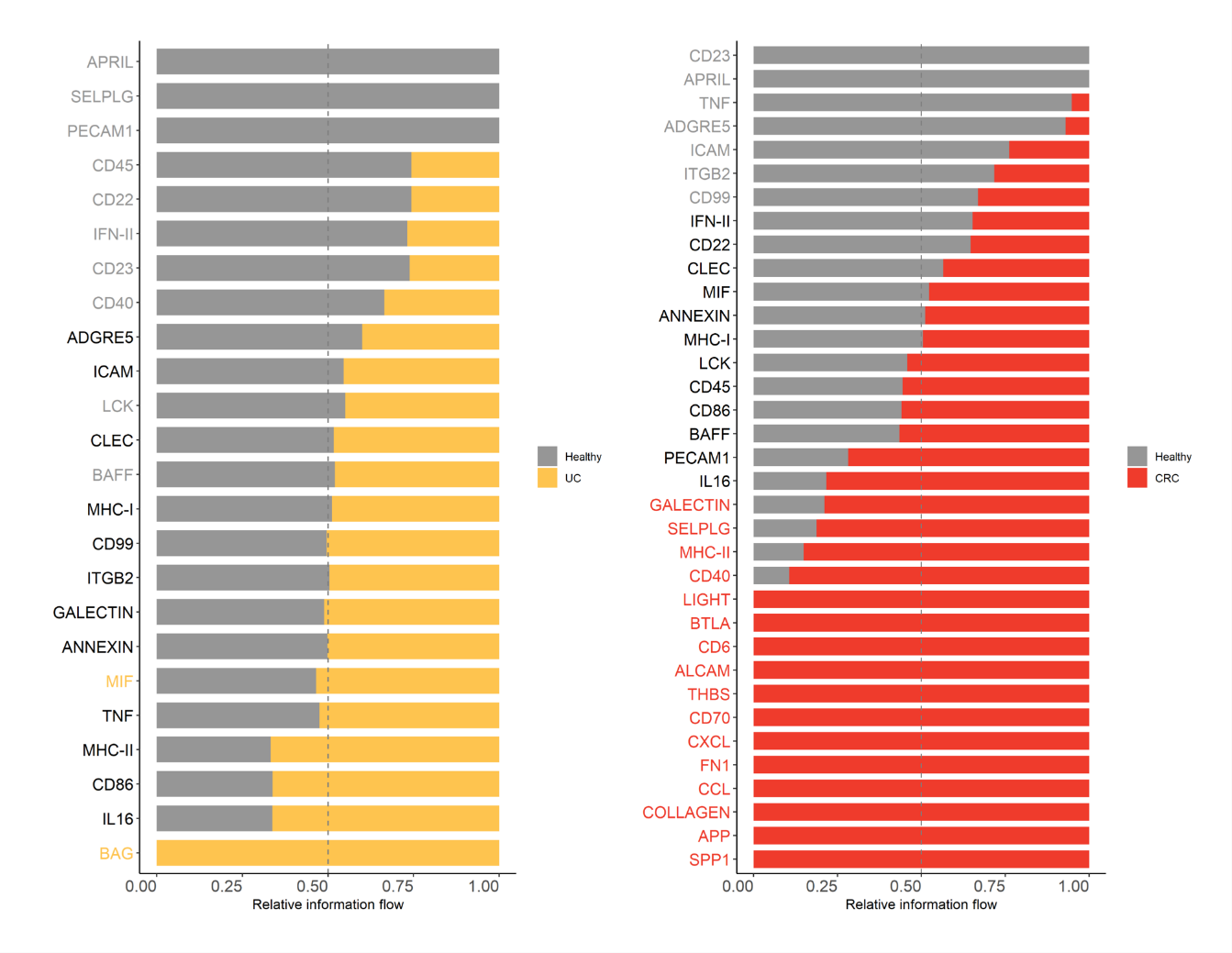
**

Supplement: S4 Fig — Relative information flow of significant signaling pathway. The color of the pathway on the y-axis means enriched pathway in the specific condition such as healthy control (grey), UC (yellow), and CRC (red). Pathway in black refers to the equally important pathway in two groups. Bar size implies the relative information flow, which is computed using the communication probability of a particular signaling pathway. The left bar plot compares the signaling pathway of healthy and UC status and the right bar plot compares healthy and CRC status. (DOCX) [file pone.0315981.s006.docx]

**S5 Fig. Biological function of *HAVCR2*+ NK cell of CRC patients**

**
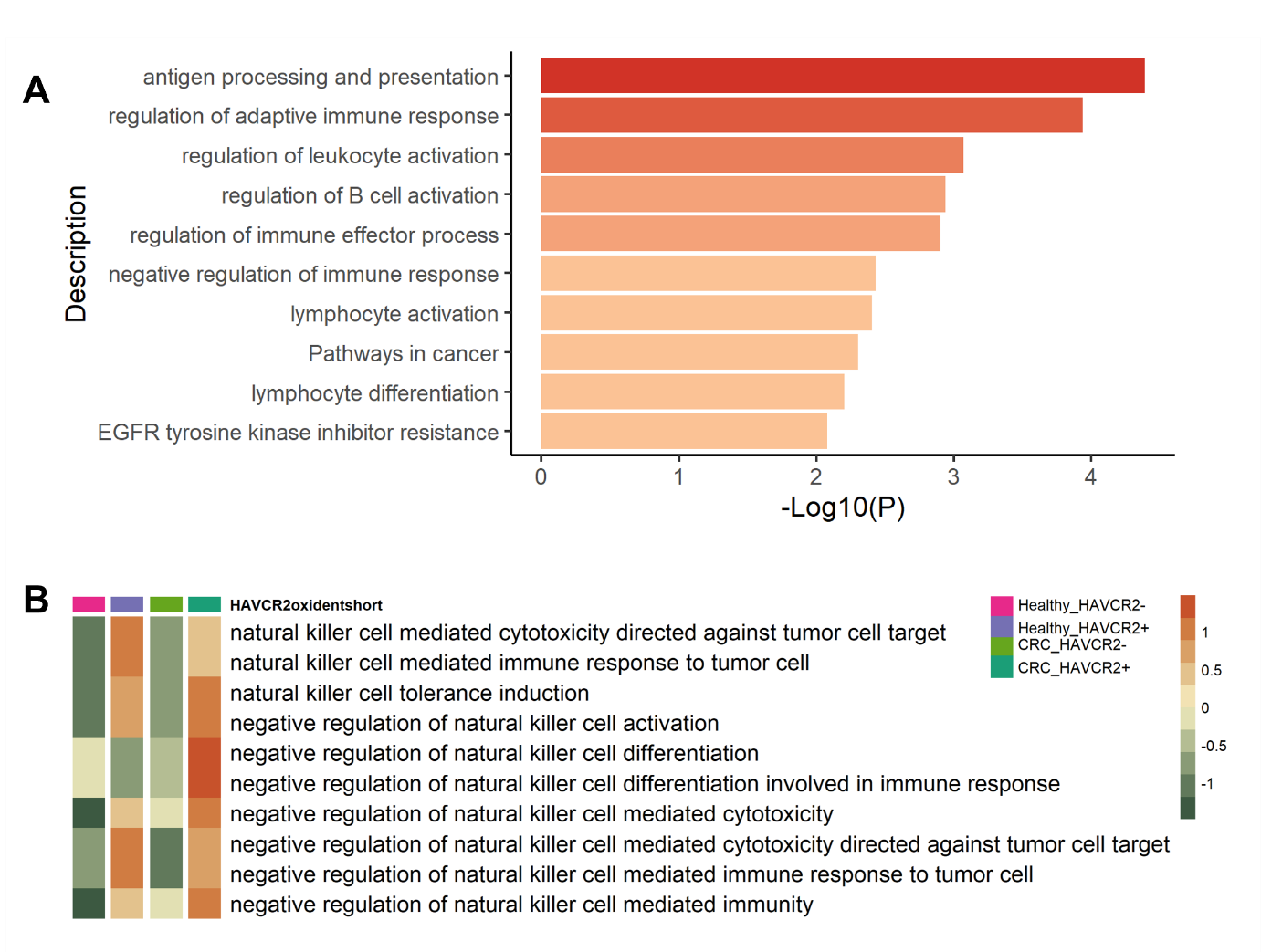
**

Supplement: S5 Fig — (A) Immunological functions of differentially coexpressed genes of the other module detected in HAVCR2+ CRC NK cells. (B) GSVA pathway for HAVCR2+ NK cell in CRC patients. Heatmaps depicted NK cell-related pathway activities depending on the presence of HAVCR2 expression in healthy and CRC groups. The colorbar describes the NES, with higher activity represented in orange and lower activity in green. (DOCX) [file pone.0315981.s007.docx]

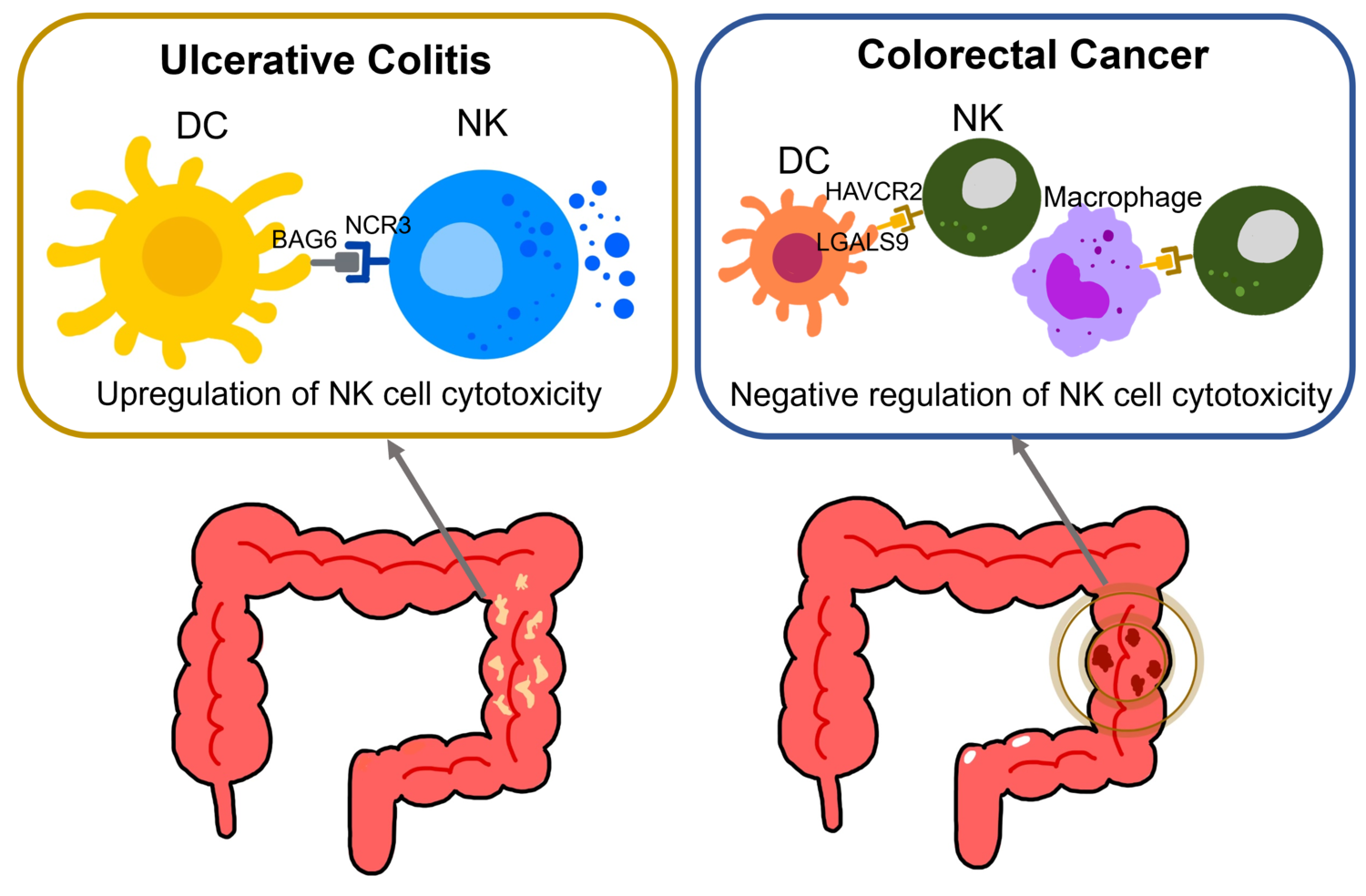

Supplement: S1 Graphical abstract — (TIFF) [file pone.0315981.s008.tiff]
